# Impact of brain frailty on language recovery in patients with acute post-stroke aphasia: a post-hoc analysis of the LEXI randomized controlled trial

**DOI:** 10.1186/s42466-026-00516-1

**Published:** 2026-07-30

**Authors:** Johannes Wischmann, Robert Forbrig, Julia Franzen, Leanna Brasch, Friederike Häfner, Katharina Feil, Lars Kellert

**Affiliations:** 1 Department of Neurology, LMU University Hospital, LMU Medizin, Ludwig-Maximilians-Universität München, Marchioninistrasse 15, 81377 Munich, Germany; 2 Institute of Neuroradiology, LMU University Hospital, LMU Medizin, Ludwig-Maximilians-Universität München, Munich, Germany; 3 Department of Neurology, University of Ulm, Ulm, Germany

**Keywords:** Stroke, Aphasia, Neurorehabilitation, Brain frailty, Randomized controlled trial

## Abstract

**Background:**

The influence of brain frailty on post-stroke aphasia recovery in the acute phase and its interaction with Speech and Language Therapy (SLT) intensity is unclear. We investigated the association between brain frailty components and language outcome and assessed whether brain frailty modifies the dose-dependent treatment effect of SLT.

**Methods:**

This is a post-hoc analysis of the LEXI multicenter randomized controlled trial, that enrolled patients with acute post-stroke aphasia from 07/2021 to 09/2024. Patients with pre-stroke cognitive impairment or dementia were not eligible for the trial. Participants were randomized to tablet-assisted SLT (Neolexon application) or standard SLT. Brain frailty was retrospectively assessed by outcome-blinded raters on interrater-adjusted baseline non-contrast CT, using cortical and subcortical atrophy, white matter changes (Fazekas score), lacunes, and chronic infarctions to derive a composite brain frailty score (BFS; range 0–3; higher scores indicate higher brain frailty burden). Primary outcome was the 90-days Bielefelder Aphasia Screening Test percentile rank.

**Results:**

56 patients (median age 75 years; 48.2% female) were included. 43 patients (76.8%) had a BFS of 0–1 and 13 (23.2%) of 2–3. Higher BFS were independently associated with worse 90-day language outcome (β -5.3; 95%CI -10.35 to -0.21; *p* = 0.042), with higher Fazekas scores contributing most (β -4.0; 95%CI -7.6 to -0.5; *p* = 0.028). Higher SLT dose was associated with improved outcome (β 0.34; 95%CI 0.07 to 0.61; *p* = 0.016). There was no statistical evidence that brain frailty modified the association between SLT duration and language outcome (p_interaction_ = 0.507), although the interaction analysis was limited by sample size.

**Conclusions:**

Higher white matter disease burden, as a component of brain frailty, was associated with poorer post-stroke language recovery in patients without pre-stroke cognitive impairment or dementia. We found no evidence that brain frailty modified the association between SLT intensity and language outcome, although larger studies are needed to assess effect modification. Routine imaging-based assessment of white matter disease burden may improve prognostic stratification and neurorehabilitation trial design.

**Clinical Trial Registration:**

ClinicalTrials.gov Identifier: NCT04080817; Study Details. Neolexon® Aphasia-App in Acute Aphasia After Stroke. ClinicalTrials.gov. Date of Registration: September 4, 2019)

**Supplementary Information:**

The online version contains supplementary material available at 10.1186/s42466-026-00516-1.

## Introduction

Successful recovery after brain injury, including acute ischemic stroke, strongly depends on individual brain reserve and the capacity to compensate acquired neurological deficits [1]. In this context, the discrepancy between neuroimaging-predicted brain age (biological age) and chronological age has emerged as a clinically relevant biomarker for clinical decision-making and prediction in the acute stroke setting prior to neurorehabilitation [2, 3]. Brain frailty as a composite construct of cerebral atrophy and vascular lesion burden is a key determinant of brain reserve and can be assessed using both MRI and non-contrast computed tomography (NCCT) [4, 5]. Individual components of brain frailty, including cortical and subcortical atrophy, conventional imaging markers of cerebral small vessel disease, and prior stroke, have been associated with worse functional outcomes including cognition after ischemic stroke and also appear to modify the treatment effect of reperfusion therapies, including intravenous thrombolysis (IVT) and endovascular treatment (ET) [6–12].

Although prior studies have linked white matter disease burden to aphasia severity, chronic language outcomes, and treatment response, the prognostic value of routinely assessable imaging markers of brain frailty in the very early phase after aphasia-causing stroke remains less well established [13, 14]. However, a higher burden of brain frailty has been associated with reduced neuroplastic potential, impaired neuronal network reorganization, and altered synaptic remodeling, which are biological mechanisms that underlie neurorehabilitation and language recovery through speech and language therapy (SLT) [15–21]. Although current stroke guidelines recommend early initiation of SLT after the index stroke, the relationships between treatment intensity, dose, timing, and patient selection remain incompletely understood, limiting the implementation of individualized rehabilitation strategies [22–24]. In this context, brain frailty may represent an underrecognized modifier of both language recovery and SLT efficacy, potentially identifying patients who require higher therapeutic intensity in the acute phase following aphasia-causing stroke.

In this study, we aimed to investigate the association between brain frailty and language recovery and to evaluate its interaction with early SLT in patients in the early acute phase following aphasia-causing stroke.

## Methods

### Study design

This study is a post-hoc analysis of the Tablet-assisted (Neolexon application) Speech and Language Therapy for Acute Post-Stroke Aphasia (LEXI) randomized controlled trial [25]. The trial was conducted between July 2021 and September 2024 at one comprehensive stroke center and two residential neurorehabilitation centers in Germany. Adult native German-speaking patients with acute post-stroke aphasia (Language Screening Test [LAST] of ≤ 13 points; range 0–15, with lower scores indicating more severe aphasia) were randomized shortly after stroke onset (median time from last seen well to randomization approximately 3 days) to receive either tablet-assisted SLT using the Neolexon application or standard SLT. Patients with pre-existing dementia or clinically apparent cognitive impairment, aphasia due to other neurological disorders, a life expectancy of ≤ 1 year, or missing written informed consent were excluded. SLT according to treatment allocation commenced immediately on the stroke unit and continued through residential neurorehabilitation, with 30 to 60-minute daily sessions delivered as individual and group therapy. Both groups were instructed in self-training: patients in the Neolexon group received individualized digital exercises via the app, while control patients used analog materials (e.g., printouts or workbooks), tailored to patient preference and ability. Logopedic and neurological assessments were performed at baseline, after 7 days (± 2 days) or at discharge (whichever occurred first), after 30 days (± 7 days) during neurorehabilitation, and after 90 days (± 14 days) during a face-to-face visit. Neuroimaging (CT or MRI) was obtained at admission to characterize stroke type and lesion location. Handedness was assessed using the Edinburgh Handedness Inventory. Reperfusion therapies (IVT and/or ET) were administered according to national and international guidelines. The primary outcome was the percentile rank in the Bielefelder Aphasia Screening Test (BIAS) 90 days post-stroke. The trial was terminated prematurely due to futility in September 2024 based on a prespecified interim analysis showing no differences in the 90-day BIAS percentile rank between both groups after enrollment of 104 patients. For this post-hoc analysis we used data from participants who (1) gave written informed consent, (3) had available baseline neuroimaging and (3) had available 90-day language assessment follow-up.

### Regulatory approvals

The original trial was registered at ClinicalTrials.gov (NCT04080817; September 4, 2019). The first patient was enrolled on July 13, 2021, and final follow-up occurred on September 7, 2024. This post hoc analysis was conducted between November 2025 and February 2026. The study protocol and statistical analysis plan for the original trial were published open access. This report followed the STROBE checklist (Supplemental Material).

### Neuroimaging assessment

Neuroimaging markers of brain frailty were retrospectively assessed on baseline NCCT by a board-certified neuroradiologist and a neurologist who were blinded to clinical outcomes and to each other’s ratings. A composite Brain Frailty Score (BFS) was calculated based on 3 imaging domains, as previously described: (1) white matter change burden, defined as a total Fazekas score (periventricular plus deep areas); (2) cerebral atrophy, defined as an intercaudate-distance-to-inner table width (CC/IT) ratio in the highest cohort-specific quartile and/or a Global Cortical Atrophy (GCA) score of 2 to 3; and (3) vascular lesions, defined as the presence of lacunes (3 to 15 mm subcortical infarcts) and/or chronic infarctions (cortical or subcortical tissue loss of > 15 mm) on axial CT [4, 5, 26, 27]. Each domain contributed 1 point, yielding a BFS range of 0 to 3. For univariable analyses, brain frailty was dichotomized into no frailty (BFS 0 to 1) and frailty (BFS 2 to 3), as done in prior studies [4, 5]. Interrater reliability was evaluated using initial independent ratings obtained prior to adjudication. Agreement for ordinal imaging variables (Fazekas and GCA scores) was assessed using weighted Cohen’s kappa statistics with linear weights, whereas agreement for binary variables (lacunes and chronic infarctions) was assessed using Cohen’s kappa. Interrater agreement was substantial to excellent for ordinal measures, with a weighted κ of 0.74 (95% CI 0.60–0.86) for the GCA score, 0.98 (95% CI 0.95-1.00) for periventricular Fazekas scores, and 0.94 (95% CI 0.88–0.98) for deep white matter Fazekas scores. Complete concordance between raters was observed for lacunes, chronic infarctions, and CC/IT ratio quartile classification (100% agreement). Disagreements in CC/IT measurements were defined as assignments to different cohort-specific quartiles. Overall disagreement rates are provided in Supplementary Table 1. All disagreements were adjudicated by a third rater blinded to outcome measures and prior ratings.

### Outcome measures

The primary outcome was language performance at 90 days post-stroke assessed using the percentile rank in the BIAS. The BIAS evaluates eight language domains and comprises 205 items, demonstrating excellent validity and reliability [28]. The BIAS in its “rehabilitation” version (BIAS-R) extends the BIAS in its “acute” version (BIAS-A) by including all of its subdomains and adding further items to assess language performance in the post-acute phases following stroke [28]. The BIAS-A was administered at baseline, and the BIAS-R at 30- and 90-day follow-up. Follow-up BIAS percentile rank scores were adjusted for baseline BIAS performance.

### Statistical analysis

Univariate analyses were performed to describe baseline characteristics and outcomes according to dichotomized brain frailty status. Continuous variables are reported as medians with interquartile ranges (IQR) and compared using Wilcoxon Rank Sum tests. Categorical variables are reported as counts and percentages and compared using χ² test. Associations between BFS and 90-day BIAS percentile rank were examined using multivariable linear regression, adjusted for age, sex, baseline National Institutes of Health Stroke Scale (NIHSS), baseline BIAS percentile rank, total SLT duration at final follow-up and randomized treatment allocation. The association between SLT duration and language recovery was evaluated using multivariable linear regression models with 90-day BIAS percentile rank as the dependent variable, adjusted for, age, sex, NIHSS and BIAS percentile rank at baseline. Treatment effect modification of SLT by brain frailty was assessed by including an interaction term between the BFS and the total SLT duration. Covariates were selected based on previously reported clinical and demographic determinants of post-stroke language recovery, including age, sex, baseline neurological stroke severity, baseline aphasia severity and SLT intensity [22, 24]. Furthermore, as a significant association of tablet-assisted SLT in the LEXI trial in patients with low NIHSS and less severe aphasia was observed, randomized treatment allocation was included as a covariate in the relevant models to account for potential effects of the trial intervention in this post-hoc association analysis. As education, assessed by years of schooling, did not differ significantly either between randomized treatment groups in the original trial or between brain frailty groups in the present analysis, it was not included as an additional covariate in the regression models to reduce the risk of overfitting given the limited sample size. Model diagnostics are provided for each model including model p-values, standard errors, variance inflation factors (VIF) and R^2^. Mediation analysis was conducted to assess whether the baseline BIAS mediated the association between BFS and 90-day language outcome. The total effect of BFS on 90-day BIAS percentile rank was estimated in a model without baseline BIAS percentile rank, while the direct effect was estimated in a model including the baseline BIAS percentile rank. The association between BFS and baseline BIAS was assessed separately. Effect estimates of mediation analysis were calculated using 1,000 bootstrap iterations. Mediation was quantified descriptively by calculating the percentage attenuation of the BFS coefficient after inclusion of the baseline BIAS. To identify which components of brain frailty contributed most to the impairment of language recovery, a component-based analysis was performed including the total Fazekas score, cerebral atrophy and vascular lesions. All components were entered simultaneously into a multivariable linear regression model adjusted for age, sex, NIHSS at baseline, baseline BIAS percentile rank, total SLT duration, and randomized treatment allocation. A sensitivity analysis was performed, including total Fazekas score only and model performance metrics were compared accordingly. All analyses were conducted in R, and statistical significance was defined as a two-sided p-value < 0.05.

**Fig. 1:**
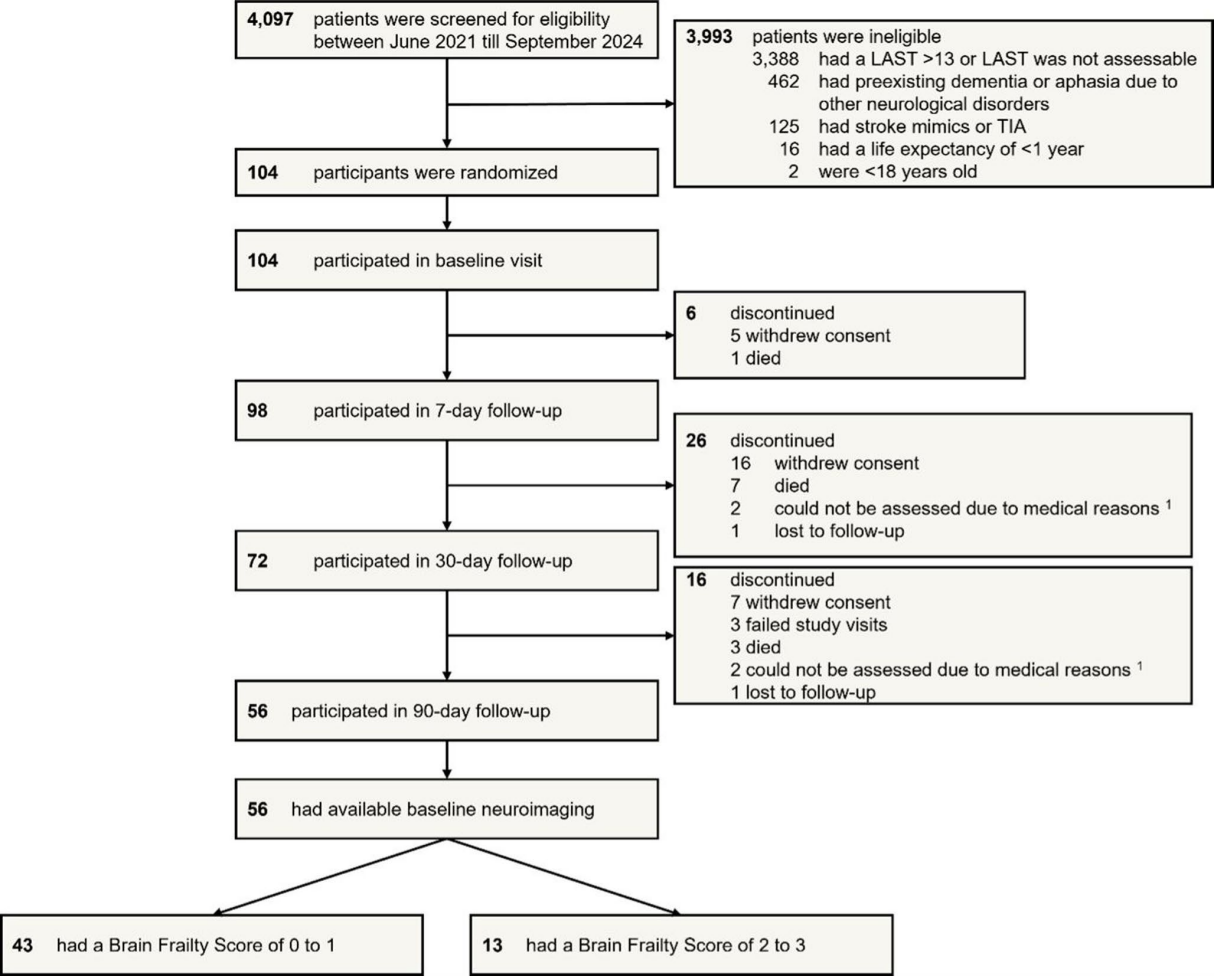
Patient inclusion flowchart of the study. ^1^medical reasons included COVID-19 infection, acute delirium, severe systemic infection and newly diagnosed brain tumor requiring surgery (one participant each). LAST Language Screening Test; TIA transient ischemic attack.

## Results

### Univariate analysis

A total of 56 patients with complete baseline imaging and 90-day language outcome data were included in the analysis (Fig. 1). Of these, 13 patients (23.2%) had a BFS of 2 to 3, and 43 patients (76.8%) had a BFS of 0 to 1 (Table 1). Patients with a BFS of 2 to 3 were numerically older (median age 80 vs. 71 years; *p* = 0.093), while sex (48.2% female vs. 51.2% female), baseline NIHSS score (median 5 vs. 4 points), baseline LAST score (median 7 vs. 7 points), and baseline BIAS percentile rank (median 25 vs. 22) were similar distributed between both groups. In the majority of patients, the qualifying acute aphasia-causing stroke was located in the left hemisphere (92.9% vs. 100.0%) due to an infarction in the middle cerebral artery territory (95.0% vs. 100.0%). Randomization to tablet-assisted SLT (53.5% vs. 53.8%) and time from last known well to randomization (3.7 days vs. 2.8 days) was similar in both groups. During the study period, both groups received a comparable total amount of SLT (median 20 h vs. 21 h), including individual SLT at the comprehensive stroke center (3.5 h vs. 3.0 h), individual SLT during neurorehabilitation (6 h vs. 7 h), and group SLT sessions during neurorehabilitation (8 h vs. 10 h). The proportion of patients performing self-training was comparable between groups (39.5% vs. 30.8%), and among those who engaged in self-training, the total training duration was not significantly different (median 5.5 h vs. 3 h). At baseline NCCT imaging, 76.9% of the patients with a BFS of 2 to 3 had a GCA score of 2 to 3 or a CC/IT ratio in the 4th quartile, compared to 14.0% of the patients with a BFS of 0 to 1 (*p* < 0.001). Chronic infarctions (69.2% vs. 16.3%) and/or lacunes (46.2% vs. 7.0%) were more frequently present in patients with a BFS of 2 to 3 compared to patients with a BFS of (*p* < 0.001). The median total Fazekas score was 3 vs. 1 in patients with brain frailty compared to patients without.

**Table 1:** Patient characteristics

Variable^a^	Total (*n* = 56)	BFS 0/1 (*n* = 43)	BFS 2/3 (*n* = 13)	*P*-value^b^
Age at randomization (years)	75 (65–82)	71 (64, 81)	80 (74, 84)	0.093
Female sex	27/56 (48.2%)	22/43 (51.2%)	5/13 (38.5%)	0.532
Prestroke modified Rankin Scale	0 (0–0)	0 (0–0)	0 (0–1)	0.227
NIHSS at baseline	5 (2–10)	5 (2–10)	4 (2–10)	0.633
Last known well to randomization (days)	3.3 (2.2–4.6)	3.7 (2.3–4.6)	2.8 (2.1–3.6)	0.219
Middle cerebral artery infarction	51/56 (91.1)	38/40 (95.0%)	13/13 (100.0%)	1.000
Left hemisphere lesioned	52/55 (94.5%)	39/42 (92.9%)	13/13 (100.0%)	1.000
Reperfusion therapy^c^	36/56 (64.3%)	30/43 (69.8%)	6/13 (46.2%)	0.186
School education (years)	11 (8–13)	11 (8–13)	12 (8–13)	0.745
Left-sided handedness	45/49 (91.8%)	36/38 (94.7%)	10/11 (90.9%)	0.542
LAST at baseline	7 (2–12)	7 (2–12)	7 (2–12)	0.757
BIAS percentile rank at baseline	24 (7–48)	25 (7–48)	22 (16–38)	0.552
Randomized to tablet-assisted SLT	30/56 (53.6%)	23/43 (53.5%)	7/13 (53.8%)	1.000
**Brain frailty neuroimaging**				
Global Cortical Atrophy Scale score				**< 0.001**
0	24/56 (42.9%)	24/43 (55.8%)	0/13 (0.0%)	
1	27/56 (48.2%)	18/43 (41.9%)	9/13 (69.2%)	
2	5/56 (8.9%)	1/43 (2.3%)	4/13 (30.8%)	
3	0/56 (0.0%)	0/43 (0.0%)	0/13 (0.0%)	1.000
CC/IT ratio	0.17 (0.14–0.20)	0.17 (0.13–0.18)	0.21 (0.17–0.22)	**0.014**
Chronic infarction present	16/56 (28.6%)	7/43 (16.3%)	9/13 (69.2%)	**< 0.001**
Chronic infarction burden (n)	0 (0–1)	0 (0–0)	1 (0–2)	**< 0.001**
Lacunes present	9/56 (16.1%)	3/43 (7.0%)	6/13 (46.2%)	**0.003**
Total Fazekas score	1 (0–2)	1 (0–2)	3 (3–4)	**< 0.001**
Fazekas score (periventricular locations)				**< 0.001**
0	22/56 (39.3%)	22/43 (51.2%)	0/13 (0.0%)	
1	23/56 (41.1%)	19/43 (44.2%)	4/ (30.8%)	
2	8/56 (14.3%)	1/43 (2.3%)	7/13 (53.8%)	
3	3/56 (5.4%)	1/43 (2.3%)	2/13 (15.4%)	
Fazekas score (deep locations)				**0.002**
0	27/56 (48.2%)	25/43 (58.1%)	2/13 (15.4%)	
1	18/56 (32.1%)	14/43 (32.6%)	4/13 (30.8%)	
2	8/56 (14.3%)	3/43 (7.0%)	5/13 (38.5%)	
3	3/56 (5.4%)	1/43 (2.3%)	2/13 (15.4%)	
**Speech and language therapy (hours)**				
Total SLT duration at final follow-up	20 (12–30)	20 (15–30)	21 (9–29)	0.712
Individual SLT in comprehensive stroke center	3.5 (2.0–5.0)	3.5 (2.0–5.0)	3.0 (2.0–4.0)	0.725
Individual SLT in neurorehabilitation	7 (5–12)	6 (5–12)	7 (6–12)	0.735
Group session SLT in neurorehabilitation	8 (5–15)	8 (5–14)	10 (5–16)	0.845
Self-training frequency	21/56 (37.5%)	17/43 (39.5%)	4/13 (30.8%)	0.747
Self-training duration	5 (0–10)	5.5 (0.0–11.0)	3.0 (0.0–7.0)	0.624
**Follow-up**				
Follow-up time (days)	99 (94–105)	100 (95–105)	98 (86–102)	0.163
BIAS percentile rank at 30 days	50 (7–77)	52 (14–81)	27 (1–54)	0.138
BIAS percentile rank at 90 days	54 (24–81)	56 (24, 92)	38 (24, 54)	0.068
Modified Rankin Scale at 90 days	1 (1–3)	1 (1–3)	3 (1–3)	0.598

Significant differences are marked bold. ^a^ n/N (%); median (IQR). ^b^ Wilcoxon Rank-Sum Test; χ² test. ^c^ intravenous thrombolysis and/or endovascular treatment.  NIHSS: National Institutes of Health Stroke Scale; LAST: Language Screening Test; BIAS: Bielefelder Aphasia Screening Test; SLT: Speech and Language Therapy. GCA: Global Cortial Atrophy; CC/IT ratio: Intercaudate-distance-to-inner table width ratio

### Brain frailty and language recovery

**Fig. 2:**
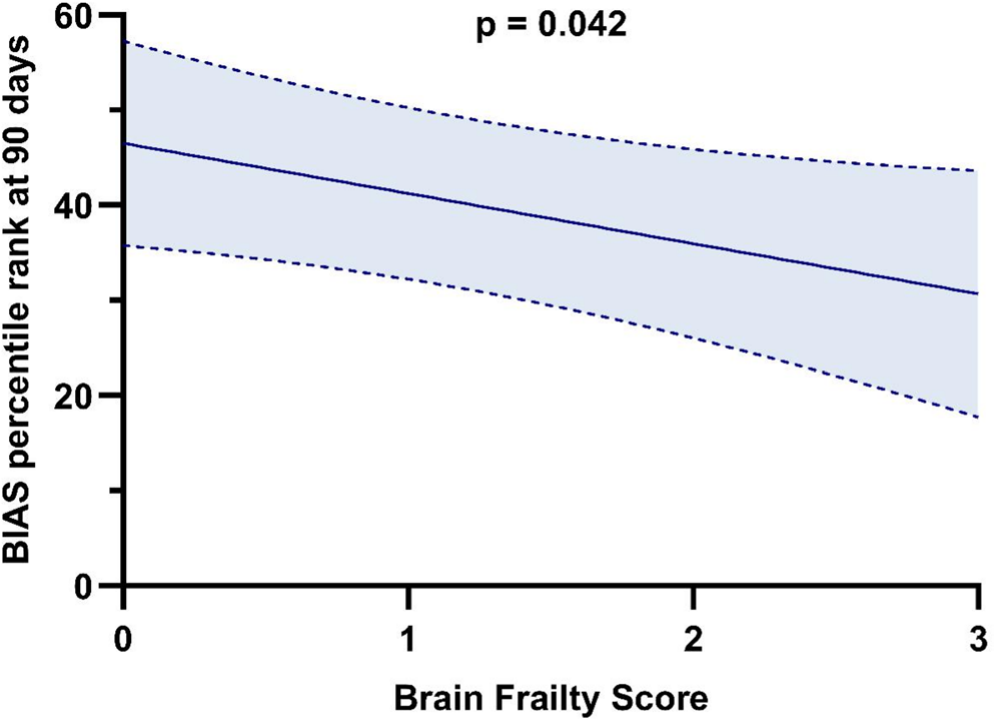
Association of brain frailty and language outcome

The median BIAS percentile rank was numerically higher in patients without brain frailty at 30 days (52 vs. 27; *p* = 0.138) and at 90 days (56 vs. 38; *p* = 0.068; Table 1). In multivariable linear regression analyses adjusted for age, sex, baseline NIHSS, baseline BIAS, total SLT duration, and randomized treatment allocation, brain frailty was independently associated with worse language outcome at 90 days (β -5.30 BIAS percentile ranks per one-point increase in the BFS; 95% CI -10.35 to -0.21; *p* = 0.042; Fig. 2; Table 2) along with higher NIHSS scores (β -2.25; 95% CI -3.26 to -1.25; p = < 0.001) and lower baseline BIAS percentage ranks (β 0.65; 95% CI 0.43 to 0.88; p = < 0.001). A mediation analysis was conducted to assess whether baseline BIAS mediated the association between BFS and 90-day BIAS (Supplemental Fig. 1). In the adjusted model without baseline BIAS, higher BFS was associated with lower 90-day BIAS (β -7.04, *p* = 0.036). After inclusion of baseline BIAS, the direct effect of BFS remained statistically significant but was attenuated (β -5.28, *p* = 0.042), corresponding to an attenuation of 25.0%. The association between BFS and baseline BIAS was not statistically significant (β -2.67, *p* = 0.412).

Adjusted association between Brain Frailty Score (BFS) and 90-day Bielefelder Aphasia Screening Test (BIAS) percentile ranks (β -5.30; 95%CI -10.35 to -0.21; *p* = 0.042). Lines represent adjusted effect estimates with 95% confidence intervals (shaded lines) stratified by BFS. The model was adjusted for, age, sex, baseline National Institutes of Health Stroke Scale scores, baseline BIAS, total speech and language therapy (SLT) duration, and randomized intervention group.

### Interaction of SLT duration, brain frailty and language recovery

**Fig. 3:**
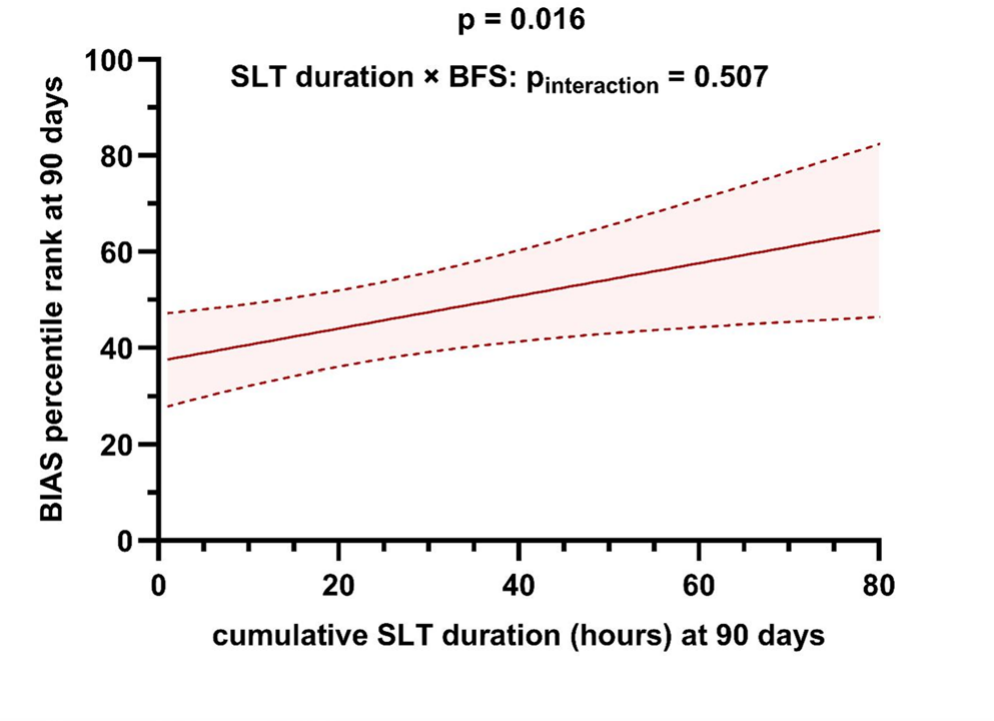
Association of speech and language therapy (SLT) duration with 90-day language outcome

In adjusted analyses including, age, sex, NIHSS and baseline BIAS each additional hour of SLT was associated with an increase of 0.34 BIAS percentile ranks at 90 days (95% CI 0.07 to 0.61; *p* = 0.016; Fig. 3; Table 3). There was no statistical evidence that the BFS modified the association between SLT duration and 90-day BIAS percentile rank (p_interaction_ = 0.507).

Adjusted association between total speech and language therapy (SLT) duration (hours) and 90-day Bielefelder Aphasia Screening Test (BIAS) percentile rank at 90 days. Predicted values were obtained from a multivariable linear regression model adjusting for, age, sex, baseline National Institutes of Health Stroke Scale scores and baseline BIAS. There was no treatment effect modification of SLT by Brain Frailty Score (BFS; p_interaction_ = 0.507). Shaded areas represent 95% confidence intervals.

### Brain frailty component analysis

**Fig. 4:**
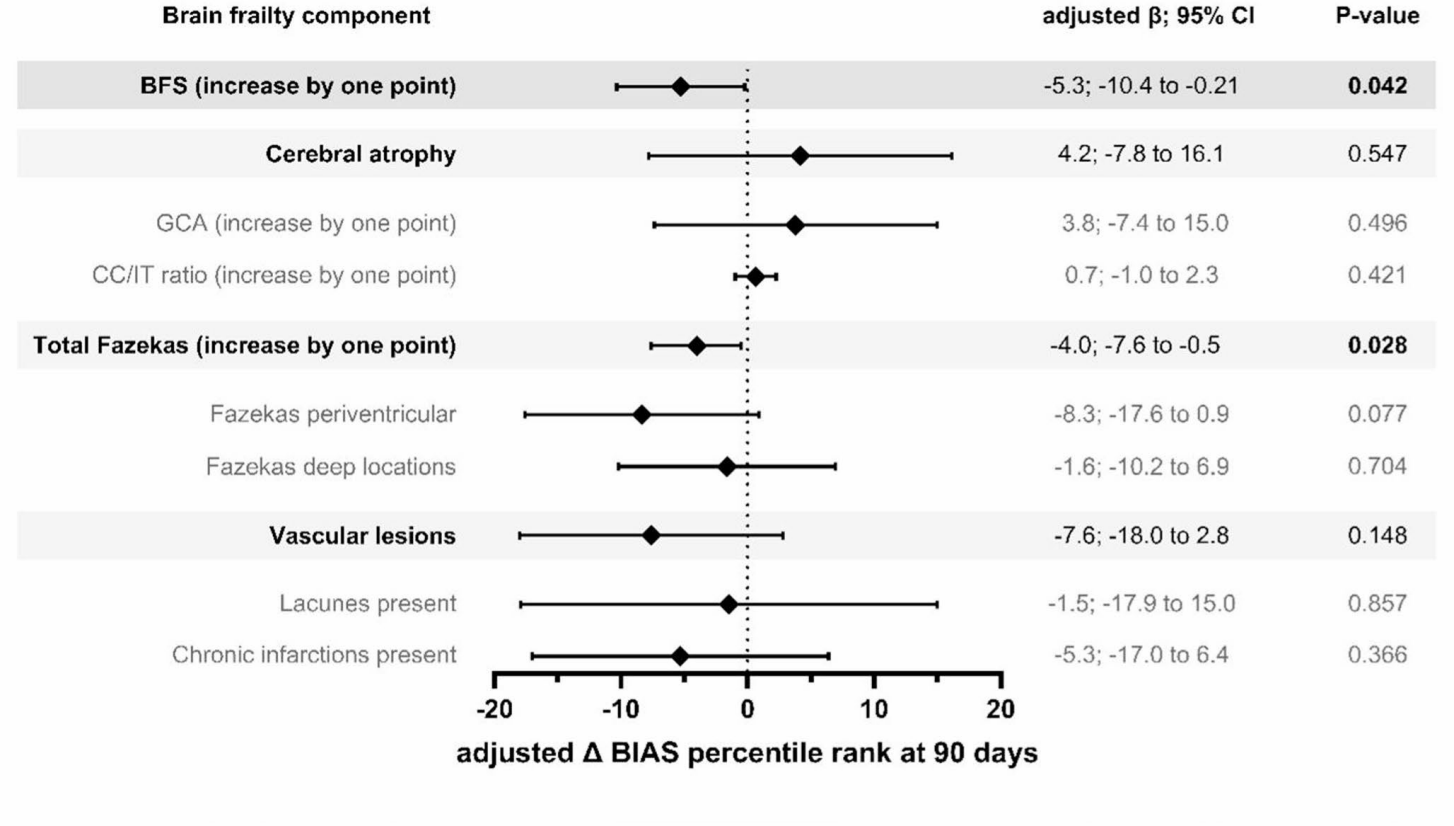
Adjusted associations between individual brain frailty neuroimaging components and language recovery at 90 days after stroke

In brain frailty component analysis, examining individual contributors to impaired language recovery, the total Fazekas score was associated with a reduction in 90-day BIAS percentile ranks (β: -4.0 BIAS percentile ranks per one point increase in the total Fazekas score, 95% CI -7.6 to -0.5; *p* = 0.028), while cerebral atrophy (β 4.2; 95% CI -7.8 to 16.1) and presence of any vascular lesions (β -7.6; 95% CI -18.0 to 2.8) were not significantly associated with a reduction in the BIAS percentile rank at 90 days. All models were adjusted for, age, sex, NIHSS, baseline BIAS, total SLT duration, and randomized treatment allocation (Fig. 4; Table 4). The sensitivity analysis, including the total Fazekas score as the single component of brain frailty into the model, yielded comparable results (β: -4.0, 95% CI -7.11 to -0.95; *p* = 0.011) and the discriminative model performance was not inferior to the full component model (R2 = 0.80 vs. 0.79) (Supplementary Table 2).

Shown are β coefficients with 95% confidence intervals (CIs) from a multivariable linear regression model assessing the independent effects of cerebral atrophy, total Fazekas score, and vascular lesions on the Bielefelder Aphasia Screening Test (BIAS) score at 90 days. Cerebral atrophy was defined as an intercaudate-to-inner table width (CC/IT) ratio in the 4th quartile and/or a Global Cortical Atrophy (GCA) scale score of 2 to 3. Vascular lesions were defined as the presence of lacunes (3–15 mm subcortical infarcts on axial CT images) and/or chronic infarctions (cortical or subcortical tissue loss > 15 mm on axial CT). The CC/IT ratio was multiplied by 100 to avoid large CIs for improved illustration. Models were adjusted for baseline BIAS score, age, sex, baseline National Institutes of Health Stroke Scale score, total duration of speech and language therapy, and randomized treatment group. Error bars indicate 95% confidence intervals, and the vertical dashed line denotes no association (β = 0).

**Table 2:** Association of Brain Frailty Score (BFS) and 90-day BIAS percentile rank

Co-variable	Estimate	95% CI	*p*-value	VIF	SE
Brain Frailty Score (per one point increase)	-5.30	-10.35 to -0.21	0.042	1.15	2.52
Age (per one year increase)	0.00	-0.45 to 0.45	0.997	1.19	0.23
Sex (female)	10.07	0.71 to 19.43	0.036	1.05	4.66
Baseline NIHSS (per one point increase)	-2.25	-3.26 to -1.25	< 0.001	1.93	0.49
Baseline BIAS (per one percentile rank increase)	0.65	0.43 to 0.88	< 0.001	1.82	0.11
Total SLT duration (per one minute increase)	0.26	-0.15 to 0.54	0.063	1.23	0.14
Randomized to intervention group (yes)	7.81	-2.26 to 17.87	0.125	1.21	5.01
**Model performance**					
*N* = 56 Model p-value: < 0.001 R^2^: 0.78					

Multivariable linear regression analysis assessing independent predictors of 90-day Bielefelder Aphasia Screening Test (BIAS) percentile ranks. Effect estimates are presented as regression coefficients with 95% confidence intervals, corresponding p values and standard errors. Variance inflation factors are shown to assess multicollinearity. Model performance metrics are reported below the table. Abbreviations: CI: Confidence interval; VIF: Variance Inflation Factor; SE: Standard Error; NIHSS: National Institutes of Health Stroke Scale; BIAS: Bielefelder Aphasia Screening Test; SLT: Speech and Language Therapy.

**Table 3:** Association of speech and language therapy (SLT) duration and 90-day language outcome

Co-variable	Estimate	95% CI	*p*-value	VIF	SE
Age (per one year increase)	-0.14	-0.60 to 0.31	0.528	1.11	0.23
Sex (female)	10.93	1.20 to 20.66	0.029	1.04	4.85
Baseline NIHSS (per one point increase)	-1.96	-2.97 to -0.95	< 0.001	1.85	0.50
Baseline BIAS (per one percentile rank increase)	0.68	0.45 to 0.91	< 0.001	1.79	0.12
Total SLT duration (per one hour increase)	0.34	0.07 to 0.61	0.016	1.11	0.14
**Model performance**					
*N* = 56 Model p-value: < 0.001 R^2^: 0.75					

Multivariable linear regression analysis assessing independent predictors of 90-day Bielefelder Aphasia Screening Test (BIAS) percentile ranks. Effect estimates are presented as regression coefficients with 95% confidence intervals, corresponding p values and standard errors. Variance inflation factors are shown to assess multicollinearity. Model performance metrics are reported below the table. Abbreviations: CI: Confidence interval; VIF: Variance Inflation Factor; SE: Standard Error; NIHSS: National Institutes of Health Stroke Scale; BIAS: Bielefelder Aphasia Screening Test; SLT: Speech and Language Therapy.

**Table 4:** Association of brain frailty components and 90-day language outcome

Co-variable	Estimate	95% CI	*p*-value	VIF	SE
Age (per one year increase)	0.09	-0.37 to 0.54	0.709	1.28	0.23
Sex (female)	13.22	3.72 to 22.72	0.007	1.15	4.72
Baseline NIHSS (per one point increase)	-2.06	-3.04 to -1.08	< 0.001	2.01	0.49
Baseline BIAS (per one percentile rank increase)	0.71	0.48 to 0.93	< 0.001	1.98	0.11
Total SLT duration (per one hour increase)	0.25	-0.02 to 0.53	0.067	1.27	0.13
Randomized to intervention group (yes)	-6.93	-16.95 to 3.11	0.171	1.27	4.98
Cerebral atrophy (yes)	4.21	-7.82 to 16.14	0.547	1.39	5.76
Total Fazekas (increase by one point)	-4.04	-7.64 to -0.46	0.028	1.70	1.78
Vascular lesions (yes)	-7.60	-18.03 to 2.84	0.148	1.27	5.18
**Model performance**					
*N* = 56 Model p-value: < 0.001 R^2^: 0.80					

Multivariable linear regression analysis assessing independent predictors of 90-day Bielefelder Aphasia Screening Test (BIAS) percentile ranks. Effect estimates are presented as regression coefficients with 95% confidence intervals, corresponding p values and standard errors. Variance inflation factors are shown to assess multicollinearity. Model performance metrics are reported below the table. Cerebral atrophy was defined as an intercaudate-to-inner table width (CC/IT) ratio in the 4th quartile and/or a Global Cortical Atrophy (GCA) scale score of 2 to 3. Vascular lesions were defined as the presence of lacunes (3–15 mm subcortical infarcts on axial CT images) and/or chronic infarctions (cortical or subcortical tissue loss > 15 mm on axial CT). Abbreviations: CI: Confidence interval; VIF: Variance Inflation Factor; SE: Standard Error; NIHSS: National Institutes of Health Stroke Scale; BIAS: Bielefelder Aphasia Screening Test; SLT: Speech and Language Therapy.

## Discussion

In this post-hoc analysis of the LEXI randomized controlled trial, we assessed the association of neuroimaging brain frailty with language recovery and its interaction with treatment responses to higher SLT doses in individuals without clinical apparent cognitive impairment or dementia. Our key findings are summarized as follows; First, higher brain frailty was independently associated with poorer language recovery following acute post-stroke aphasia, even after adjustment for age, baseline language impairment, and stroke severity. Each one-point increase in the BFS was associated with a reduction of approximately 5 points in the 90-day BIAS percentile rank score. Component-based and sensitivity analyses suggested that this association was primarily driven by white matter disease burden, and that the composite BFS did not provide incremental prognostic value over total Fazekas score alone in this cohort. Second, greater SLT intensity was associated with improved language outcomes independent of stroke severity, and this effect appears to not be attenuated by a higher baseline white matter hyperdensity burden. Together, these results suggest that although higher white matter disease burden identifies patients at increased risk for impaired recovery, it does not appear to diminish the therapeutic benefit of intensified SLT.

These findings may be clinically relevant given that known predictors, including age, baseline aphasia and stroke severity account for only approximately half of the observed variance in chronic aphasia outcomes, suggesting that additional neurobiological factors like a reduced brain reserve may influence language recovery trajectories [18, 23]. Furthermore, in this study brain frailty was not associated with baseline language impairment and the effect of baseline brain frailty on 90-day language outcome was not mediated by baseline language impairment in the present study, consistent with previous reports [29]. This pattern suggests that stroke severity may primarily determine initial aphasia severity, whereas the integrity of the remaining brain tissue may play a more prominent role in post-stroke language recovery. Neuroimaging-based frailty measures are not routinely incorporated into acute stroke assessments, nor are they commonly used for stratification or covariate adjustment in aphasia rehabilitation trials. However, brain frailty represents a pragmatic imaging marker of premorbid brain health that captures vascular and neurodegenerative burden beyond stroke-specific characteristics and offers potential prognostic value [2, 30]. Because brain frailty can be assessed on routine NCCT imaging, its integration into clinical prognostication, patient counseling, and trial design should therefore be considered.

We observed, that the association between brain frailty and language recovery appeared to be largely driven by higher Fazekas scores, in line with previous studies suggesting that white matter hyperintensity burden is associated with poorer language performance and language outcomes in the chronic post-stroke phase [18, 31, 32]. Experimental and connectome-based work further suggests that diffuse white matter damage reduces compensatory network efficiency, providing a plausible biological mechanism linking vascular brain injury to impaired recovery capacity [14, 16, 29, 31–33]. In contrast, we did not observe an independent association between cortical atrophy and language recovery. Although neurodegenerative imaging markers, including frontotemporal atrophy, have been associated with worse baseline language function and reduced recovery after aphasia-causing stroke, the absence of such an effect in our cohort is likely explained by the exclusion of patients with clinically relevant cognitive impairment and dementia in the original trial [25, 34–36]. This interpretation is supported by the low prevalence of advanced global cortical atrophy in our population compared to other studies, with approximately 91% of patients exhibiting GCA grades 0 to1, 9% grade 2, and no patients with grade 3 atrophy [4, 5]. Because patients with clinically apparent cognitive impairment or dementia were excluded from the LEXI trial, our findings are therefore not generalizable to these populations and a conclusion weather the composite BFS is superior over white matter disease grading alone cannot be drawn from the present data. Consequently, the prognostic value of baseline brain frailty in patients with acute post-stroke aphasia, including individuals with pre-existing cognitive impairment or dementia warrants further investigation.

Two prior studies suggested, that right hemispheric white matter hyperintensity burden predicts response to language therapy independent of lesion-related factors, and that higher baseline periventricular white matter disease is associated with reduced responsiveness to neuromodulatory interventions such as transcranial direct current stimulation [37, 38]. Our findings differ from these reports, as we did not observe statistical evidence that brain frailty and the burden of white matter disease in particular modified the association between SLT duration and 90-day language outcome. Several factors may explain this discrepancy: First, prior studies assessed white matter disease in more anatomically specific regions, including right-hemispheric or periventricular white matter hyperintensity burden, whereas our analysis used a global imaging-based brain frailty construct and total Fazekas score derived from routine baseline NCCT. Second, previous studies largely focused on chronic post-stroke aphasia or specific therapeutic interventions, whereas the present cohort was assessed in the acute phase after stroke and received early rehabilitation as part of a pragmatic trial setting. Third, total SLT duration in the present analysis was not randomized or protocolized, but reflected naturally occurring variation in therapy exposure. Finally, the interaction analysis was limited by sample size, particularly the small number of patients with higher brain frailty. Therefore, the absence of a statistically significant interaction cannot be interpreted as evidence that white matter disease or brain frailty does not influence treatment responsiveness. Larger studies with randomized or protocolized therapy dose are needed to determine whether brain frailty modifies the effect of SLT intensity. Interestingly, female sex was consistently associated with higher BIAS percentile ranks at 90 days across all models. This finding does not appear to be attributable to differences in baseline stroke severity, age, or baseline BIAS percentile rank in the original trial, which were similarly distributed between men and women. However, given the limited sample size, residual confounding or chance cannot be excluded. The relatively low intensity of SLT in the original trial, reflecting clinical practice in the acute and early rehabilitation setting, may have contributed, as previous evidence suggests that female patients may benefit most from lower-intensity therapy within the first months after aphasia onset [24]. This finding supports the concept that non-lesional patient-related factors may influence language recovery and highlights the need to further investigate sex-specific differences, including potential interactions with white matter disease burden and therapy intensity.

Strengths of this study include the use of data derived from a randomized controlled trial. In addition, the BIAS served as the primary outcome measure and provides a comprehensive assessment of speech and language function with excellent sensitivity, specificity, and interrater reliability [28]. This study has limitations; First, the sample size was limited due to premature termination of the original trial [25], which may limit generalizability and more granular analyses of brain frailty components, such as distinguishing between periventricular and deep leukoaraiosis as potential differential drivers of impaired language recovery. Furthermore, the sample size limits statistical power and stability of reported effect estimates and the results should therefore be considered exploratory. Second, NCCT rather than MRI was used to assess imaging markers of brain frailty as a pragmatic approach; however, prior studies have demonstrated good agreement between NCCT- and MRI-based measures [27]. Third, in the original trial, SLT doses beyond tablet-assisted SLT were not randomized, potentially introducing bias by indication.

## Conclusion

Higher white matter disease burden was associated with poorer language recovery after acute post-stroke aphasia in individuals without pre-stroke cognitive impairment. We found no statistical evidence that brain frailty modified the association between SLT duration and language outcome; however, the analysis was underpowered to exclude clinically relevant effect modification. Given that white matter disease burden can be readily assessed on routine baseline neuroimaging in acute stroke care, its integration into clinical practice may improve prognostic stratification and support individualized rehabilitation planning. Furthermore, consideration of white matter disease burden should be incorporated as a potential confounder in neurorehabilitation trials.

## Supplementary Information

Below is the link to the electronic supplementary material.

Supplementary Material 1

## Data Availability

Anonymized data not published within this article will be made available by request from any qualified investigator.
