# Supplementary material for: Impact of brain frailty on language recovery in patients with acute post-stroke aphasia: a post-hoc analysis of the LEXI randomized controlled trial
Source: Neurol Res Pract. 2026 Jul 30;8(1):58. doi: 10.1186/s42466-026-00516-1 (PMC13425768; doi:10.1186/s42466-026-00516-1)
Supplement: Supplementary file 1 — Supplementary Material 1 [file 42466_2026_516_MOESM1_ESM.docx]

SUPLLEMENTAL MATERIAL

| **Content** | **Page** |
| --- | --- |
| Supplementary Table 1: Interrater agreement for brain frailty imaging variables. | 2 |
| Supplementary Fig. 1: Mediation analysis of the association between brain frailty and language outcome. | 3 |
| Supplementary Table 2: Association of total Fazekas score and 90-day language outcome (sensitivity analysis). | 4 |
| STROBE check list | 5 |
| Study Group | 8 |

Supplementary Table 1: Interrater agreement for brain frailty imaging variables.

| **Imaging parameter** | **Conflicts** | **Interrater Agreement** | **Point Estimate** | **95% Confidence Interval** |
| --- | --- | --- | --- | --- |
| Global Cortical Atrophy score | 11 | 80.4% | 0.74 | 0.60-0.86 |
| Fazekas score (periventricular locations) | 3 | 94.6% | 0.98 | 0.95-1.00 |
| Fazekas score (deep locations) | 5 | 91.1% | 0.94 | 0.88-0.98 |
| Lacunes (present/absent) | 0 | 100% | NA | NA |
| Chronic infarctions (present/absent) | 0 | 100% | NA | NA |
| CC/IT ratio (quartile classification) | 0 | 100% | NA | NA |

Weighted Cohen’s kappa statistics were calculated for ordinal variables using linear weights. Reliability analyses were based on independent ratings before adjudication. Percent agreement is reported for variables with complete concordance between raters, for which kappa or intraclass correlation coefficients cannot be reliably estimated.

Supplementary Fig. 1: Mediation analysis of the association between brain frailty and language outcome. **
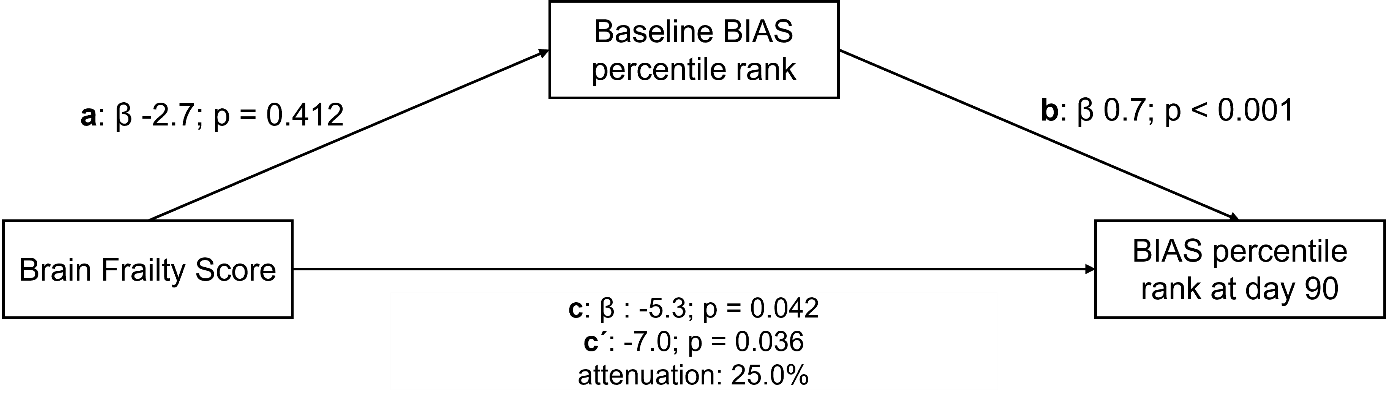
**

Causal mediation analysis illustrating the relationship between Brain Frailty Score (BFS), baseline Bielefelder Aphasia Screening Test (BIAS), and language outcome at 90 days after stroke. Path **a** represents the association between BFS and baseline BIAS, path **b** represents the association between baseline BIAS and 90-day BIAS adjusted for BFS, and path **c** represents the direct effect of BFS on 90-day BIAS after adjustment for baseline BIAS. The total effect **c´** represents the unadjusted association between BFS and 90-day BIAS. Effect estimates of mediation analysis were calculated using 1,000 bootstrap iterations. Values shown correspond to standardized regression coefficients with associated p values from adjusted linear regression models, adjusted for age, sex, National Institutes of Health Stroke Scale at baseline, total speech and language therapy duration and randomized treatment group. The indirect (mediated) effect was not statistically significant, indicating that baseline language severity did not significantly mediate the association between brain frailty and language recovery.

Supplementary Table 2: Association of total Fazekas score and 90-day language outcome (sensitivity analysis).

| **Co-variable** | **Estimate** | **95% CI** | **p-value** | **VIF** | **SE** |
| --- | --- | --- | --- | --- | --- |
| Age (per one year increase) | 0.09 | -0.36 to 0.55 | 0.688 | 1.27 | 0.23 |
| Sex (female) | 12.70 | 3.54 to 21.87 | 0.008 | 1.06 | 4.56 |
| Baseline NIHSS (per one point increase) | -2.06 | -3.01 to -1.11 | < 0.001 | 1.87 | 0.47 |
| Baseline BIAS (per one percentile rank increase) | 0.70 | 0.49 to 0.92 | < 0.001 | 1.80 | 0.11 |
| Total SLT duration (per one hour increase) | 0.29 | 0.02 to 0.55 | 0.039 | 1.23 | 0.13 |
| Randomized to intervention group (yes) | -6.08 | -16.00 to 3.84 | 0.224 | 1.23 | 4.94 |
| Total Fazekas (increase by one point) | -4.03 | -7.11 to -0.95 | 0.011 | 1.24 | 1.53 |
| **Model performance** | | | | | |
| Model p-value: < 0.001  R^2^: 0.79 | | | | | |

Multivariable linear regression analysis assessing independent predictors of 90-day Bielefelder Aphasia Screening Test (BIAS) percentile ranks. Effect estimates are presented as regression coefficients with 95% confidence intervals, corresponding p values and standard errors. Variance inflation factors are shown to assess multicollinearity. Model performance metrics are reported below the table. Abbreviations: CI: Confidence interval; VIF: Variance Inflation Factor; SE: Standard Error; NIHSS: National Institutes of Health Stroke Scale; BIAS: Bielefelder Aphasia Screening Test; SLT: Speech and Language Therapy.

STROBE checklist

|  | Item No | Recommendation | Page  No |
| --- | --- | --- | --- |
| **Title and abstract** | 1 | (*a*) Indicate the study’s design with a commonly used term in the title or the abstract | 1 |
|  |  | (*b*) Provide in the abstract an informative and balanced summary of what was done and what was found | 2 |
| Introduction | | | |
| Background/rationale | 2 | Explain the scientific background and rationale for the investigation being reported | 4 |
| Objectives | 3 | State specific objectives, including any prespecified hypotheses | 4 |
| Methods | | | |
| Study design | 4 | Present key elements of study design early in the paper | 5 |
| Setting | 5 | Describe the setting, locations, and relevant dates, including periods of recruitment, exposure, follow-up, and data collection | 5 |
| Participants | 6 | (*a*) *Cohort study*—Give the eligibility criteria, and the sources and methods of selection of participants. Describe methods of follow-up  *Case-control study*—Give the eligibility criteria, and the sources and methods of case ascertainment and control selection. Give the rationale for the choice of cases and controls  *Cross-sectional study*—Give the eligibility criteria, and the sources and methods of selection of participants | 5 |
|  |  | (*b*) *Cohort study*—For matched studies, give matching criteria and number of exposed and unexposed  *Case-control study*—For matched studies, give matching criteria and the number of controls per case |  |
| Variables | 7 | Clearly define all outcomes, exposures, predictors, potential confounders, and effect modifiers. Give diagnostic criteria, if applicable | 6, 7 |
| Data sources/ measurement | 8 | For each variable of interest, give sources of data and details of methods of assessment (measurement). Describe comparability of assessment methods if there is more than one group | 6, 7 |
| Bias | 9 | Describe any efforts to address potential sources of bias | 7, 8 |
| Study size | 10 | Explain how the study size was arrived at | 5 |
| Quantitative variables | 11 | Explain how quantitative variables were handled in the analyses. If applicable, describe which groupings were chosen and why | 7 |
| Statistical methods | 12 | (*a*) Describe all statistical methods, including those used to control for confounding | 7 |
|  |  | (*b*) Describe any methods used to examine subgroups and interactions | 7 |
|  |  | (*c*) Explain how missing data were addressed | 7 |
|  |  | (*d*) *Cohort study*—If applicable, explain how loss to follow-up was addressed  *Case-control study*—If applicable, explain how matching of cases and controls was addressed  *Cross-sectional study*—If applicable, describe analytical methods taking account of sampling strategy | 7 |
|  |  | (*e*) Describe any sensitivity analyses | 7 |
| Results | | | |
| Participants | 13 | (a) Report numbers of individuals at each stage of study—eg numbers potentially eligible, examined for eligibility, confirmed eligible, included in the study, completing follow-up, and analysed | Fig. 1 |
|  |  | (b) Give reasons for non-participation at each stage | Fig. 1 |
|  |  | (c) Consider use of a flow diagram | Fig. 1 |
| Descriptive data | 14 | (a) Give characteristics of study participants (eg demographic, clinical, social) and information on exposures and potential confounders | Table 1 |
|  |  | (b) Indicate number of participants with missing data for each variable of interest | Table 1 |
|  |  | (c) *Cohort study*—Summarise follow-up time (eg, average and total amount) | 9 |
| Outcome data | 15 | *Cohort study*—Report numbers of outcome events or summary measures over time | 9 |
|  |  | *Case-control study—*Report numbers in each exposure category, or summary measures of exposure |  |
|  |  | *Cross-sectional study—*Report numbers of outcome events or summary measures |  |
| Main results | 16 | (*a*) Give unadjusted estimates and, if applicable, confounder-adjusted estimates and their precision (eg, 95% confidence interval). Make clear which confounders were adjusted for and why they were included | Table 2-4 |
|  |  | (*b*) Report category boundaries when continuous variables were categorized | Table 2-4 |
|  |  | (*c*) If relevant, consider translating estimates of relative risk into absolute risk for a meaningful time period |  |
| Other analyses | 17 | Report other analyses done—eg analyses of subgroups and interactions, and sensitivity analyses | 16 |
| Discussion | | | |
| Key results | 18 | Summarise key results with reference to study objectives | 21 |
| Limitations | 19 | Discuss limitations of the study, taking into account sources of potential bias or imprecision. Discuss both direction and magnitude of any potential bias | 23 |
| Interpretation | 20 | Give a cautious overall interpretation of results considering objectives, limitations, multiplicity of analyses, results from similar studies, and other relevant evidence | 22 |
| Generalisability | 21 | Discuss the generalisability (external validity) of the study results | 23 |
| Other information | | | |
| Funding | 22 | Give the source of funding and the role of the funders for the present study and, if applicable, for the original study on which the present article is based | 24 |

LEXI Investigators (study group).

| **First name** | **Last name** | **Affiliation** |
| --- | --- | --- |
| Johannes | Wischmann | Department of Neurology, LMU University Hospital Munich, Munich, Germany |
| Leanna | Brasch | Department of Neurology, LMU University Hospital Munich, Munich, Germany |
| Julia | Franzen | Department of Neurology, LMU University Hospital Munich, Munich, Germany |
| Jennifer | Schwierz | Department of Neurology, LMU University Hospital Munich, Munich, Germany |
| Oksana | Kovalenko | Department of Neurology, LMU University Hospital Munich, Munich, Germany |
| Andrea | Gutmann | Institute for Medical Information Processing, Biometry and Epidemiology (IBE), Faculty of Medicine, LMU Munich, Munich, Germany  Department of Anaesthesiology, LMU University Hospital, Munich, Germany |
| Franziska | Erbert | Department of Neurology, LMU University Hospital Munich, Munich, Germany  Department of Orthopedics and Trauma Surgery, Musculoskeletal University Center Munich (MUM), LMU University Hospital Munich, Munich, Germany |
| Luisa | Bußmann | Department of Neurology, LMU University Hospital Munich, Munich, Germany  Department of Orthopedics and Trauma Surgery, Musculoskeletal University Center Munich (MUM), LMU University Hospital Munich, Munich, Germany |
| Julia | Lauer | Department of Neurology, LMU University Hospital Munich, Munich, Germany  Department of Orthopedics and Trauma Surgery, Musculoskeletal University Center Munich (MUM), LMU University Hospital Munich, Munich, Germany |
| Claudia | Dumberger | Department of Neurology, LMU University Hospital Munich, Munich, Germany  Department of Orthopedics and Trauma Surgery, Musculoskeletal University Center Munich (MUM), LMU University Hospital Munich, Munich, Germany |
| Alexander | Mandl | Department of Neurology, LMU University Hospital Munich, Munich, Germany  Department of Orthopedics and Trauma Surgery, Musculoskeletal University Center Munich (MUM), LMU University Hospital Munich, Munich, Germany |
| Marie | Mehringer | Department of Neurology, LMU University Hospital Munich, Munich, Germany  Department of Orthopedics and Trauma Surgery, Musculoskeletal University Center Munich (MUM), LMU University Hospital Munich, Munich, Germany |
| Annette | Münsterer | Department of Neurology, LMU University Hospital Munich, Munich, Germany  Department of Orthopedics and Trauma Surgery, Musculoskeletal University Center Munich (MUM), LMU University Hospital Munich, Munich, Germany |
| Julia | Spitzer | Department of Neurology, LMU University Hospital Munich, Munich, Germany  Department of Orthopedics and Trauma Surgery, Musculoskeletal University Center Munich (MUM), LMU University Hospital Munich, Munich, Germany |
| Lena | Winterhalter | Department of Neurology, LMU University Hospital Munich, Munich, Germany  Department of Orthopedics and Trauma Surgery, Musculoskeletal University Center Munich (MUM), LMU University Hospital Munich, Munich, Germany |
| Vanessa | Frank | Department of Neurology, LMU University Hospital Munich, Munich, Germany  Department of Orthopedics and Trauma Surgery, Musculoskeletal University Center Munich (MUM), LMU University Hospital Munich, Munich, Germany |
| Marika | Rheinwald | Department of Neurology, LMU University Hospital Munich, Munich, Germany  Department of Orthopedics and Trauma Surgery, Musculoskeletal University Center Munich (MUM), LMU University Hospital Munich, Munich, Germany |
| Katharina | Lehner | Institute for Social Sciences and Humanities, Ostbayerische Technische Hochschule OTH, Regensburg, Germany |
| Franziska | Ammer | Clinic for Neurology, Medical Park, Reithof Park, Bad Feilnbach, Germany |
| Angelika | Pfahler | Clinic for Neurology, Medical Park, Reithof Park, Bad Feilnbach, Germany |
| Stefanie | Lampart | Clinic for Neurology, Medical Park, Reithof Park, Bad Feilnbach, Germany |
| Charlotte | Young | Clinic for Neurology, Medical Park, Reithof Park, Bad Feilnbach, Germany |
| Peter | Young | Clinic for Neurology, Medical Park, Reithof Park, Bad Feilnbach, Germany |
| Barbara | Goettert | Rehabilitation Center, Passauer Wolf, Bad Griesbach, Germany |
| Marcella | Bitzan | Rehabilitation Center, Passauer Wolf, Bad Griesbach, Germany |
| Stephanie | Rinder | Rehabilitation Center, Passauer Wolf, Bad Griesbach, Germany |
| Oliver | Meier | Rehabilitation Center, Passauer Wolf, Bad Griesbach, Germany |
| Katharina | Feil | Department of Neurology, University of Ulm, Ulm, Germany |
| Lars | Kellert | Department of Neurology, LMU University Hospital Munich, Munich, Germany |
